# Screening of whole genome sequences identified high-impact variants for stallion fertility

**DOI:** 10.1186/s12864-016-2608-3

**Published:** 2016-04-14

**Authors:** Rahel Schrimpf, Maren Gottschalk, Julia Metzger, Gunilla Martinsson, Harald Sieme, Ottmar Distl

**Affiliations:** Institute for Animal Breeding and Genetics, University of Veterinary Medicine Hannover, Bünteweg 17p, 30559 Hannover, Germany; State Stud Celle of Lower Saxony, Spörckenstraße 10, 29221 Celle, Germany; Clinic for Horses, Unit for Reproduction Medicine, University of Veterinary Medicine Hannover, Bünteweg 15, 30559 Hannover, Germany

**Keywords:** Whole genome screening, High-impact variants, Stallion fertility, NOTCH1

## Abstract

**Background:**

Stallion fertility is an economically important trait due to the increase of artificial insemination in horses. The availability of whole genome sequence data facilitates identification of rare high-impact variants contributing to stallion fertility. The aim of our study was to genotype rare high-impact variants retrieved from next-generation sequencing (NGS)-data of 11 horses in order to unravel harmful genetic variants in large samples of stallions.

**Methods:**

Gene ontology (GO) terms and search results from public databases were used to obtain a comprehensive list of human und mice genes predicted to participate in the regulation of male reproduction. The corresponding equine orthologous genes were searched in whole genome sequence data of seven stallions and four mares and filtered for high-impact genetic variants using SnpEFF, SIFT and Polyphen 2 software. All genetic variants with the missing homozygous mutant genotype were genotyped on 337 fertile stallions of 19 breeds using KASP genotyping assays or PCR-RFLP. Mixed linear model analysis was employed for an association analysis with de-regressed estimated breeding values of the paternal component of the pregnancy rate per estrus (EBV-PAT).

**Results:**

We screened next generation sequenced data of whole genomes from 11 horses for equine genetic variants in 1194 human and mice genes involved in male fertility and linked through common gene ontology (GO) with male reproductive processes. Variants were filtered for high-impact on protein structure and validated through SIFT and Polyphen 2. Only those genetic variants were followed up when the homozygote mutant genotype was missing in the detection sample comprising 11 horses. After this filtering process, 17 single nucleotide polymorphism (SNPs) were left. These SNPs were genotyped in 337 fertile stallions of 19 breeds using KASP genotyping assays or PCR-RFLP. An association analysis in 216 Hanoverian stallions revealed a significant association of the splice-site disruption variant g.37455302G>A in NOTCH1 with the de-regressed estimated breeding values of the paternal component of the pregnancy rate per estrus (EBV-PAT). For 9 high-impact variants within the genes CFTR, OVGP1, FBXO43, TSSK6, PKD1, FOXP1, TCP11, SPATA31E1 and NOTCH1 (g.37453246G>C) absence of the homozygous mutant genotype in the validation sample of all 337 fertile stallions was obvious. Therefore, these variants were considered as potentially deleterious factors for stallion fertility.

**Conclusions:**

In conclusion, this study revealed 17 genetic variants with a predicted high damaging effect on protein structure and missing homozygous mutant genotype. The g.37455302G>A NOTCH1 variant was identified as a significant stallion fertility locus in Hanoverian stallions and further 9 candidate fertility loci with missing homozygous mutant genotypes were validated in a panel including 19 horse breeds. To our knowledge this is the first study in horses using next generation sequencing data to uncover strong candidate factors for stallion fertility.

**Electronic supplementary material:**

The online version of this article (doi:10.1186/s12864-016-2608-3) contains supplementary material, which is available to authorized users.

## Background

A major challenge in current equine genomics is to understand the genetic basis of mutations influencing stallion fertility. In Hanoverian stallions, several studies implicated loci that have been shown to play a significant role in stallion fertility. Mutations within CRISP3 [1, 2], SPATA1, INHBA, ACE, SP17, FSHB, PRLR [3–5], PLCz1 [6] and FKBP6 [7] were significantly associated with stallion fertility. Abnormalities in number of X- and/or Y chromosomes have been shown as causes for abnormal sexual development [8–12]. Until now, only few mutations were identified to be associated with sub- or infertility in stallions. FKBP6 genotyping was recommended for stallions affected by impaired acrosome reaction (IAR). In human, only 20 % of male factor infertility can be diagnosed using genetic testing [13]. Whole-genome sequencing approaches facilitate a comprehensive identification of high-impact variants with potentially deleterious effects on traits analysed [14]. Rare genetic variants are more likely to be functionally deleterious and to cause loss-of function of coding proteins [14, 15]. Recently, whole-genome sequencing was used to uncover the roles of rare genetic variants associated with spermatogenic failure in men [16]. To date, no whole-genome sequence screens were performed for stallion fertility. We employed next-generation sequence (NGS) data to screen male fertility associated genes in order to unravel the role of genetic variants for which damaging effects on the protein structure were predicted. Herein, we employed bioinformatic analysis to identify human and mice genes linked through common gene ontology (GO) male reproductive processes. In order to focus the search for rare high-impact variants within the gene list, we filtered the whole genomes sequence data for variants which were only present as wild type homozygotes or heterozygotes in fertile horses. We assumed that in fertile stallions, variants with deleterious effects on fertility should be restricted to heterozygous genotypes for the recessive alleles or the homozygous wild type genotype for the dominant allele, and the presence of one of these variants may reduce stallion fertility. The objective of the present study was to genotype rare high-impact variants retrieved from NGS-data of 11 horses in order to unravel harmful genetic variants in a large sample of stallions.

## Results

### Search for genes involved in stallion reproduction

Gene ontology (GO) terms for male reproductive processes and Ensembl and NCBI databases were used to search for genes with an effect on male fertility in human and mice. We retrieved a list of 1256 genes identities (Additional file 1). Using g:Profiler [17, 18] we could convert 1194 genes to known equine orthologs and had to exclude 62 genes due to unknown equine annotation or unknown chromosomal position. Using PANTHER classification system [19, 20], the 1194 male fertility related genes were assigned to 67 pathways, 85 molecular functions, 82 biological processes and 16 cellular components.

### Mutation detection in 1194 equine genes

We searched the whole genome sequences of all 11 horses for genetic variants within the 1194 male reproduction related genes. The first filter sorted out all genetic variants for which both homozygous genotypes and the heterozygous genotypes were present. This filter left 1259 genetic variants with influence on the coding sequence and contained only variants for which the mutant homozygous genotype was not present. The second filtering for high impact variants retrieved a total of 19 SNPs within 18 genes and 38 indels within 31 genes (Additional file 2). Further evaluation was restricted to SNP variants only. Of the 19 SNPs, 16 SNPs were located in 15/1194 genes for stallion reproduction and three SNPs (ENSECAG00000020135, ENSECAG00000021286 and ENSECAG00000018118) were not obviously related to male reproduction, but located within uncharacterized genes or pseudogenes on the complementary DNA strand (Table 1, Additional file 3). The most frequently predicted high-impact effects were donor splice site disruptions (*n* = 7) and stop codon gains (*n* = 6) (Table 2). We could assign these 15 genes with possibly rare high-impact variants to four pathways, 14 molecular functions, 14 biological processes and one cellular component. Using GeneMANIA, we constructed a gene-gene functional network for 13/15 genes based on GO annotation (Additional file 4).

**Table 1:** Comparison of SNPs with high effect on protein structure in genes for stallion fertility among breeds using whole-genome sequences of fertile stallions

SNP ID	Chrom	Gene	Arabian stallion*	Sorraia stallion*	Hanoverian stallion*	Hanoverian stallion*	Dülmen Horse mare*	Icelandic stallion**	Przewalski stallion**	Standard-bred stallion**	Norwegian fjord mare**	Arabian mare*	‘Twilight‘ mare**
g.26775767G>C	ECA1	NEURL1	0/0	0/0	**0/1**	0/0	**0/1**	0/0	0/0	0/0	0/0	0/0	0/0
g.77472655G>C	ECA3	KDR	0/0	**0/1**	0/0	**0/1**	0/0	0/0	0/0	0/0	0/0	0/0	0/0
g.74610774C>T	ECA4	CFTR	0/0	0/0	0/0	0/0	0/0	0/0	**0/1**	0/0	0/0	0/0	0/0
g.56937215C>T	ECA5	OVGP1	0/0	0/0	0/0	**0/1**	0/0	0/0	0/0	0/0	0/0	0/0	0/0
g.45985131A>G	ECA9	FBXO43	0/0	0/0	0/0	0/0	**0/1**	0/0	0/0	0/0	0/0	0/0	0/0
g.82699661C>T	ECA9	TSSK6	0/0	0/0	0/0	0/0	**0/1**	0/0	0/0	0/0	0/0	0/0	0/0
g.7083659A>T	ECA11	SLC9A3R1	0/0	0/0	**0/1**	**0/1**	0/0	0/0	0/0	**0/1**	0/0	0/0	0/0
g.40694339G>A	ECA13	PKD1	0/0	0/0	0/0	0/0	0/0	**0/1**	0/0	0/0	0/0	0/0	0/0
g.6704968C>T	ECA16	GHRL	0/0	0/0	0/0	0/0	0/0	0/0	0/0	0/0	**0/1**	0/0	0/0
g.19034281C>T	ECA16	FOXP1	0/0	0/0	0/0	0/0	**0/1**	0/0	0/0	0/0	0/0	0/0	0/0
g.21894180G>A	ECA17	FNDC3A	**0/1**	0/0	0/0	0/0	**0/1**	0/0	0/0	**0/1**	0/0	0/0	0/0
g.32635273T>C	ECA20	BTNL2	0/0	0/0	**0/1**	0/0	0/0	0/0	0/0	0/0	**0/1**	0/0	0/0
g.35255390T>C	ECA20	TCP11	0/0	0/0	0/0	0/0	0/0	**0/1**	0/0	0/0	0/0	0/0	0/0
g.4323852G>A	ECA25	SPATA31E1	0/0	0/0	0/0	0/0	0/0	0/0	0/0	0/0	**0/1**	0/0	0/0
g.37453246G>C	ECA25	NOTCH1	0/0	0/0	0/0	0/0	0/0	0/0	0/0	0/0	0/0	**0/1**	0/0
g.37455302G>A	ECA25	NOTCH1	0/0	**0/1**	**0/1**	0/0	0/0	0/0	0/0	0/0	0/0	0/0	0/0
g.79813487A>T	ECA3	ENSECAG00000020135	0/0	0/0	0/0	0/0	0/0	**0/1**	0/0	0/0	0/0	0/0	0/0
g.25184403G>C	ECA7	ENSECAG00000021286	0/0	0/0	0/0	0/0	0/0	**0/1**	0/0	0/0	0/0	0/0	0/0
g.30073089G>A	ECA13	ENSECAG00000018118	0/0	0/0	0/0	**0/1**	0/0	0/0	0/0	0/0	0/0	0/0	0/0

The variant ID (SNP ID), chromosomal position (ECA), gene symbol (Gene) and the horse genotypes are given. Genotypes either represent wild type variant (0/0), or mutant in heterozygote state (0/1), highlighted in bold. The animals marked with an asterisk were sequenced by Metzger et al. (2014) [52] the animals marked with two asterisks were sequenced by Orlando et al. (2013) [51]. The reference horse genome (Twilight) is derived from the Thoroughbred mare Twilight (EquCab2.70)

**Table 2:** Identification of variants with high effect on protein structure in genes for stallion fertility

SNP ID	Accession no.	Gene	Location in gene	High SNP effect	Substitution type
g.26775767G>C	ss1457622628	NEURL1	Exon 5	Stop gained	p.S155*
g.77472655G>C	ss1457622630	KDR	Intron 30	Splice site donor	
g.74610774C>T	ss1457622632	CFTR	Exon 17	Stop gained	p.Q894*
g.56937215C>T	ss1457622634	OVGP1	Exon 6	Stop gained	p.R145*
g.45985131A>G	ss1457622636	FBXO43	Exon 1	Start lost	p.M1T
g.82699661C>T	ss1457622637	TSSK6	Exon 3	Stop gained	p.W155*
g.7083659A>T	ss1457622638	SLC9A3R1	Splice site intron 9	Splice site donor	
g.40694339G>A	ss1457622640	PKD1	Exon 17	Stop gained	p.W339*
g.6704968C>T	ss1457622641	GHRL	Splice site intron 1	Splice site acceptor	
g.19034281C>T	ss1457622642	FOXP1	Splice site intron 4	Splice site donor	
g.21894180G>A	ss1457622643	FNDC3A	Splice site intron 27	Splice site acceptor	
g.32635273T>C	ss1457622644	BTNL2	Exon 1	Start lost	p.M1T
g.35255390T>C	ss1457622645	TCP11	Exon 1	Start lost	p.M1T
g.4323852G>A	ss1457622646	SPATA31E1	Exon 3	Stop gained	p.R423*
g.37453246G>C	ss1457622647	NOTCH1	Splice site intron 4	Splice site acceptor	
g.37455302G>A	ss1457622648	NOTCH1	Splice site intron 14	Splice site donor	
g.79813487A>T	ss1457622631	ENSECAG00000020135	n.a.	Splice site acceptor,	
				Splice site donor	
g.25184403G>C	ss1457622635	ENSECAG00000021286	n.a.	Splice site donor	
g.30073089G>A	ss1457622639	ENSECAG00000018118	n.a.	Splice site donor	

The variant ID (SNP ID) and dbSNP accession number, gene symbol and location in the gene, type of high-impact effect and corresponding substitution type on protein level are given. Variants causing a stop codon are marked with an asterix

### Validation of high-impact SNPs in fertile stallions

In total, we genotyped 19 SNPs whereof two SNPs, one in FNDC3A and one SNP on the complementary DNA strand (ENSECAG00000018118), proved monomorphic in a sample of 96 stallions. Out of the 17 remaining SNPs genotyped, 15 SNPs were related to male reproduction. Two SNPs (ENSECAG00000020135, ENSECAG00000021286) were located within uncharacterized genes/pseudogenes on the complementary DNA strand whereas these SNPs had no high impact within the candidate genes on protein structure. Genotyping for the 17 polymorphic SNPs was performed in 337 stallions from 19 different breeds using PCR-RFLP and competitive allele specific PCR (KASP) genotyping technique (Additional file 5). Three SNPs within NEURL1, GHRL and BTNL2 deviated from Hardy Weinberg equilibrium (HWE) (Table 3). For all SNPs, the minor allele was represented by the mutant allele with a mean minor allele frequency (MAF) of 0.07 (range = 0.002–0.306). For 6/17 variants all three genotypes were found, of which the mutant homozygous genotypes were underrepresented with a mean genotype frequency of 0.06 (range = 0.003–0.223). For 9/17 genetic variants, no stallion exhibited the mutant homozygous genotype (Table 4). The expected number of mutant homozygous genotypes for stallions, calculated as q^2^ (mutant allele frequency squared) x number of stallions (n) stallions, revealed two variants within FOXP1 and OVGP1 with expected number of genotypes of 0.92 and 1.04 (Table 5). The variants within ENSECAG00000020135 and ENSECAG00000021286 were homozygous wild type in all stallions (Table 4).

**Table 3:** Validation of 17 high-impact SNPs in 337 fertile stallions

		HWE			
Polymorphism	Candidate gene	HET	*X* ^2^	DF	P-HWE
g.26775767G>C	NEURL	0.116	22.094	1	<0.0001
g.77472655G>C	KDR	0.228	0.324	1	0.569
g.74610774C>T	CFTR	0.004	0.001	1	0.976
g.56937215C>T	OVGP1	0.111	0.810	1	0.368
g.45985131A>G	FBXO43	0.032	0.082	1	0.775
g.82699661C>T	TSSK6	0.068	0.380	1	0.538
g.7083659A>T	SLC9A3R1	0.398	0.476	1	0.490
g.40694339G>A	PKD1	0.013	0.013	1	0.911
g.6704968C>T	GHRL	0.019	18.572	1	<0.001
g.19034281C>T	FOXP1	0.104	0.959	1	0.327
g.32635273T>C	BTNL2	0.168	120.250	1	<0.0001
g.35255390T>C	TCP11	0.022	0.040	1	0.842
g.4323852G>A	SPATA31E1	0.009	0.007	1	0.933
g.37453246G>C	NOTCH1	0.023	0.040	1	0.841
g.37455302G>A	NOTCH1	0.226	0.272	1	0.602
g.79813487A>T	ENSECAG00000020135	0	0	0	–
g.25184403G>C	ENSECAG00000021286	0	0	0	–

For each polymorphism, corresponding candidate gene, heterozygosity (HET), test for Hardy Weinberg equilibrium (HWE) with the respective chi square (*X* ^2^) statistic, degrees of freedom (DF) and *P*-value (P-HWE) are given

**Table 4:** Minor allele (MA), minor allele frequencies (MAF), minor genotype frequencies (MAG) for high-impact variants within candidate genes for stallion fertility validated in 337 fertile stallions

	Allele		Genotype		
Polymorphism	MA	MAF	AA	AB	BB
g.26775767G>C	C	0.0865	0.855	0.116	0.028
NEURL1			272/318	37/318	9/318
g.77472655G>C	C	0.1266	0.759	0.228	0.013
KDR			243/320	73/320	4/320
g.74610774C>T	T	0.0018	0.997	0.004	0
CFTR			283/284	1/284	
g.56937215C>T	T	0.0556	0.889	0.111	0
OVGP1			208/234	26/234	
g.45985131A>G	G	0.0158	0.968	0.032	0
FBXO43			306/316	10/316	
g.82699661C>T	T	0.0338	0.933	0.068	0
TSSK6			290/311	21/311	
g.7083659A>T	T	0.2919	0.509	0.398	0.093
SLC9A3R1			164/322	128/322	30/322
g.40694339G>A	A	0.0062	0.988	0.013	0
PKD1			317/321	4/321	
g.6704968C>T	T	0.0125	0.978	0.019	0.003
GHRL			314/321	6/321	1/321
g.19034281C>T	T	0.0522	0.896	0.104	0
FOXP1			283/316	33/316	
g.32635273T>C	C	0.3064	0.610	0.168	0.223
BTNL2			200/328	55/328	73/328
g.35255390T>C	C	0.0110	0.978	0.022	0
TCP11			310/317	7/317	
g.4323852G>A	A	0.0047	0.991	0.009	0
SPATA31E1			318/321	3/321	
g.37453246G>C	C	0.0113	0.978	0.023	0
NOTCH1			304/311	7/311	
g.37455302G>A	A	0.1341	0.753	0.226	0.021
NOTCH1			247/328	74/328	7/328
g.79813487A>T	T	0	1	0	0
ENSECAG00000020135			74/74		
g.25184403G>C	C	0	1	0	0
ENSECAG00000021286			74/74

**Table 5:** Evaluation of high-impact variants for stallion fertility

SNP ID	GENE	EBV-PAT association	GT_missing_	2pq_high_	2pq_low_	E (q^2^_stallion_)	Number of breeds	Private Group I	Rare MAF < 0.01 Group II	MAF 0.01-0.1 Group III	MAF >0.1 Group IV
g.26775767G>C	NEURL						7			+	
g.77472655G>C	KDR						11				+
g.74610774C>T	CFTR		+		0.004	0.001	2		+		
g.56937215C>T	OVGP1		+	0.111		0.723	3			+	
g.45985131A>G	FBXO43		+		0.032	0.079	4			+	
g.82699661C>T	TSSK6		+	0.068		0.355	8			+	
g.7083659A>T	SLC9A3R1						12				+
g.40694339G>A	PKD1		+		0.013	0.012	2		+		
g.6704968C>T	GHRL						5			+	
g.19034281C>T	FOXP1		+	0.111		0.861	11			+	
g.32635273T>C	BTNL2						7				+
g.35255390T>C	TCP11		+		0.022	0.038	2			+	
g.4323852G>A	SPATA31E1		+		0.009	0.007	3		+		
g.37453246G>C	NOTCH1		+		0.023	0.040	4			+	
g.37455302G>A	NOTCH1	0.008					9				+
g.79813487A>T	ENSECAG00000020135						1	+			
g.25184403G>C	ENSECAG00000021286						1	+			

Significant associations of the high-impact variants with the estimated breeding values of the paternal component for the pregnancy rate per estrus (EBV-PAT) in Hanoverian stallions (*n* = 216) are indicated by Bonferroni-corrected *P*-values. For each variant, a plus sign indicates the missing mutant homozygous genotype (GT_missing_), high and low heterozygous genotype frequencies (2pq_high,_ 2pq_low_) and the expected number of mutant homozygous genotypes for stallions E (q^2^_stallion_) are given. The number of breeds where the variants were observed is counted and a grouping (group I-IV) by the minor allele frequency (MAF) is given

We grouped the genetic variants genotyped into four groups (I-IV) according to their minor allele frequency (MAF). In group I, the mutant allele did not occur (MAF = 0), in group II, the mutant allele occurred in a very low frequency, which were classified as rare variants (MAF < 0.01) [14]. In group III, the mutant allele occurred in a low frequency (MAF > 0.01–0.1), and in group IV the mutant allele had a moderate frequency (MAF > 0.1). Variants in non-male reproduction related genes ENSECAG00000020135 and ENSECAG00000021286 were sorted into group I. Variants in CFTR, PKD1 and SPATA31E1 were classified as rare variants (group II). Eight variants were sorted into group III and four variants into group IV (Table 5). The distribution of the variants genotyped within discovery breeds and validation cohorts should allow distinguish between private SNPs and SNPs shared by several breeds. With the exception of two private Icelandic SNPs within ENSECAG00000020135 and ENSECAG00000021286, all high-impact SNPs were shared by 2–12 breeds (Additional file 6).

### Association analysis in Hanoverian stallions

In 216 Hanoverian stallions, a nominally significant association with de-regressed EBV-PAT was found for the NOTCH1 variant g.37455302G>A (*P* = 0.00003) using a model fitting the genotypes. Accounting for multiple testing of 15 SNPs using Bonferroni correction, resulted in a significant association for the NOTCH1 variant g.37455302G>A (*P* = 0.00045) explaining 11 % of the variation of de-regressed EBV-PAT. A mixed model analysis accounting for genomic relationships among stallions gave a *P*-value of 0.00052 and after Bonferroni correction a *P*-value of 0.00828. Hanoverian stallions exhibiting the wild-type for the NOTCH1-associated SNP had a de-regressed EBV-PAT of 101 onto the scale 100 ± 20 indicating stallion fertility closely at the population average. The differences among the NOTCH1-homozygotes were 41 points of the de-regressed EBV-PAT (*P*-value < 0.0001). Stallions heterozygous for the mutant NOTCH1-variant had a mean of 100 for the de-regressed EBV-PAT. Other variants failed the nominal significance thresholds (Additional file 7). Stallions heterozygous or homozygous for the KDR-variant showed a higher mean of de-regressed EBV-PAT than homozygous wild type stallion. Due to high standard errors and low genotype frequencies, no significant associations, but a tendency among the homozygous and heterozygous genotype means for de-regressed EBV-PAT were found for six SNPs within NEURL1, OVGP1, FOXP1, NOTCH1 (g.37453246G>C), SPATA31E1 and FBXO43 (Additional file 7).

### Classification of high-impact variants

Based on validation and association results, we distinguished three classes among the high-impact variants (Table 6). The first class (class I) regards loci with a significant association with EBV-PAT and a low to moderate allele frequency. Only the NOTCH1 variant g.37455302G>A fulfills these requirements for class I loci. The second class (class II) should contain loci which may be considered as putative indicators for fertility. Herein, we classified 10 variants as class II loci. The third class (class III) comprises high-impact variants with no obvious significant relation to stallion reproduction. As class III loci we sorted in two private SNPs, only present in Icelandic stallions and variants with all three genotypes but not significantly associated with de-regressed EBV-PAT.

**Table 6:** Most promising high-impact variants in genes contributing to stallion fertility

Loci for stallion fertility		Loci for stallion infertility		No obvious relation to stallion reproduction			
Class I		Class II		Class III			
SNP ID	Gene	SNP ID	Gene	SNP ID	Gene	SNP ID	Gene
g.37455302G>A	NOTCH1	g.74610774C>T	CFTR	g.79813487A>T	ENSECAG00000020135	g.32635273T>C	BTNL2
		g.56937215C>T	OVGP1	g.25184403G>C	ENSECAG00000021286	g.7083659A>T	SLC9A3R1
		g.45985131A>G	FBXO43			g.6704968C>T	GHRL
		g.82699661C>T	TSSK6			g.26775767G>C	NEURL1
		g.40694339G>A	PKD1			g.77472655G>C	KDR
		g.19034281C>T	FOXP1				
		g.35255390 T>C	TCP11				
		g.4323852G>A	SPATA31E1				
		g.37453246G>C	NOTCH1				

Three classes of high-impact variants with corresponding single nucleotide polymorphism (SNP) identities (ID) and gene are given

## Discussion

Screening of whole genome sequences for high-impact genetic variants within gene networks for male fertility in stallions appeared useful to identify variants that may highly influence stallion fertility. All high-impact variants we classified due to stop codon gains, start losses, donor or acceptor splice site disruptions can be classified as loss- of-function (LOF) mutations [14, 21]. A major effect on fertility by loss-of-function mutations was demonstrated in previous studies [22–25]. Data from this study indicate a high-impact variant as a stallion fertility-associated locus (class I) in NOTCH1 and 9 high-impact variants as putative stallion fertility-associated loci (class II). Genes putatively associated with stallion fertility included CFTR, OVGP1, FBXO43, TSSK6, PKD1, FOXP1, TCP11, SPATA31E1 and NOTCH1 (g.37453246G>C). These genes are connected with each other via functional interacting networks based on GO male fertility processes.

In mice, NOTCH1 gain-of-function resulted in reduced male fertility due to failure of spermatogenesis [26], while immotile spermatozoa and sterility was detected in NEURL1 null mice [27]. The fertile stallions used for validation were frequently uses in artificial insemination, thus sperm motility and morphology are in the normal range in these stallions. Fertile stallions that exhibited unfavorable genotypes in NOTCH1-high-impact variants had de-regressed EBV-PAT as well EBV-PAT below the population mean suggesting reduced fertility due to other unknown damaging mechanism on sperm function. For the KDR high-impact variant g.77472655G>C in KDR, heterozygous or homozygous carriers showed putatively higher de-regressed EBV-PAT and EBV-PAT implicating a beneficial effect of this variant. The latter is supported by the ‘less-is-more’ hypothesis [28] and an advantageous effect of deleterious variants due to positive selection [29]. An advantageous effect on reproduction of deleterious variants alleles due to positive selection was recently demonstrated in buffalo [30]. NOTCH1, NEURL1 and KDR are key factors for male fertility, making their application for stallion fertility highly desirable. NOTCH1 is part of the Notch signaling network and regulates interactions between physically adjacent cells [31]. NEURL1 is required for axonemal integrity in spermatozoa [27]. It functionally belongs to spermatogenesis (GO:0007283) and is associated to spermatid differentiation (GO:0048515), sperm motility (GO:0030317) and spermatid differentiation (GO:0048515). KDR is present in spermatozoa [32] and was identified as a regulator of germ cell survival during establishment of spermatogenesis in cattle [33].

Based on our hypothesis of missing mutant genotypes in fertile stallions, we detected 9 putative loci for stallion fertility defined as class II variants. These non-private and high-impact variants were only present in very low to or low frequency. The expected frequency of mutant homozygous genotypes for stallions E(q^2^_stallion_) indicated a low-probability of their occurrence in fertile stallions. As an explanation for the missing homozygous mutant genotypes in fertile stallions one can assume that homozygous mutant genotypes are limited to infertile stallions. This may be further tested in an unselected sample of male foals under the assumption that these mutations are still segregating in very few males with the homozygous mutant genotype may be infertile.

Class II variants within CFTR and TSSK6 indicated high-impact on male fertility, confirmed by several studies in human and mice. In infertile men, high-effect variants at acceptor splice site in CFTR were found in non-obstructive azoospermia, oligospermia, asthenospermia and teratospermia men [34, 35]. Infertility due to spermatogenic impairment was reported in male TSSK6 knockout mice [36] and in Chinese men exhibiting a triallelic SNP in TSSK6 [37]. PKD1 is required for male reproductive tract development. PKD1-null mice develop multiple organ defects in the male reproductive tract and infertility [38]. TCP-11 modulates sperm fertilizing ability. It stimulates capacitation and inhibits spontaneous acrosome loss [39, 40]. Subfertility in human could result from alterations in the structure of TCP-11 [39].

Validation of class II high-impact variants within SPATA31E1, OVGP1, FOXP1, FBXO43 indicate further putative loci for stallion fertility, however the consequences of mutations on male infertility in human and mice are not yet known. Nevertheless, SPATA31E1 functionally belongs to spermatogenesis (GO:0007283). OVGP1 is linked to single fertilization (GO:0007338) via binding to oocyte zona pellucida [41]. It enhances sperm viability, motility [42] and capacitation and shows beneficial effects on fertilization and early embryo development [43, 44]. FOXP1 is an androgen-responsive transcription factor that modulates androgen receptor signaling [45]. FBXO43 place a role in sperm-induced meiotic exit [46] and adjustment of the number of round spermatids [47].

## Conclusions

Out of 1194 equine genes involved in male fertility processes we identified the g.37455302G>A NOTCH1 variant as a high-impact SNP with a significant effect on Hanoverian stallion fertility. As this detected high-impact-variant only occurs in a rare frequency in fertile stallions of many different breeds we recommend selection against this unfavourable genotype to improve stallion fertility. Next, we identified 9 high-impact SNPs in CFTR, OVGP1, FBXO43, TSSK6, PKD1, FOXP1, TCP11, SPATA31E1 and NOTCH1 (g.37453246G>C) absent in fertile stallions with putative deleterious effects in fertility of stallions. As a consequence, an early identification of carriers in unselected young stallions should be a beneficial to select only high-quality stallions for future breeding.

## Methods

### Ethics statement

All animal work has been conducted according to the national and international guidelines for animal welfare. The Lower Saxony state veterinary office at the Niedersächsisches Landesamt für Verbraucherschutz und Lebensmittelsicherheit, Oldenburg, Germany, was the responsible Institutional Animal Care and Use Committee (IACUC) for this specific study. The EDTA-blood sampling for the present study had been approved by the IACUC of Lower Saxony, the state veterinary office Niedersächsisches Landesamt für Verbraucherschutz und Lebensmittelsicherheit, Oldenburg, Germany (registration number 33.42502-05-07A482).

### Search for genes effecting stallion reproduction

Gene ontology (GO) terms related to male reproduction were used to obtain a comprehensive list of known human und mice genes predicted to participate in the regulation of male reproduction. We used the go.obo download file which contains core GO ontology terms provided by the gene ontology consortium (http://purl.obolibrary.org/obo/go.obo, accessed 02.08.2014). In addition, a keyword search for annotated genes in the genome browsers Ensembl and NCBI for ‘*male reproduction’*, ‘*male infertility’*, *‘male gonad development’,* ‘*spermatogenesis’*, ‘*acrosome reaction'*, was conducted. We then searched NCBI PubMed with keywords for mutations within genes affecting male reproduction in human and livestock animals (including horses) from articles published before November 1st 2014. We employed Ensembl BioMart [48, 49] (http://www.ensembl.org/biomart/martview/) to retrieve all human genes (Ensembl gene ids) associated with a GO term and converted human genes into equine orthologs using g.Profiler [17, 18] (http://biit.cs.ut.ee/gprofiler/gconvert.cgi). If an equine orthologous gene was not available in Ensembl we searched for uncharacterized novel equine orthologs based on human annotations from the Ensemble genome browser (http://www.ensembl.org/index.html) or searched for annotations matching the NCBI genome database (http://www.ncbi.nlm.nih.gov/). For equine annotations, chromosomes (ECA) and gene start and stop positions in base pairs (bp) were extracted using Ensembl BioMart. Genes were mapped to GO annotations (molecular function biological process, cellular component) using PANTHER (Protein analysis through evolutionary relationships, version 8.0) database [19, 20] (http://www.pantherdb.org). The database GeneMania [50] (http://www.genemania.org/) was used to visualise co-expression and common pathways for networks of male fertility related genes.

### Detection of deleterious mutations

We screened publicly available whole genome sequences of seven stallions including each one Icelandic, Standardbred and Przewalski stallion [51], two Hanoverian stallions and each one Arabian and Sorraia stallion [52], and four mares including each one Arabian, Norwegian fjord [51], Dülmen Horse [52], and the re-sequenced reference genome of the Thoroughbred mare Twilight [51] for mutations within selected candidate genes for stallion fertility.

Variants within candidate genes were extracted according to the equine chromosomal positions obtained from the Ensembl annotation. We excluded all genetic variants with hits in the genome databases dbSNP (ftp://ftp.ncbi.nih.gov/snp/organisms/horse_9796/chr_rpts/, download 19.09.2013), Broad Institute (http://www.broadinstitute.org/ftp/distribution/horse_snp_release/v2/*.xls, download 19.09.2013) and Ensembl (ftp://ftp.ensembl.org/pub/release-73/variation/gvf/equus_caballus/Equus_caballus.gvf.gz, download 25.07.2014).

Functional analysis of variants obtained from re-sequencing data was performed using SnpEFF software [53]. The SnpEFF impact category HIGH was used to filter SNPs for putative high-impact effects (large chromosome deletion, exon deletion, insertion/deletion frame shift, donor splice site disruptions, acceptor splice site disruptions, stop codon gains, stop codon losses, start losses). Validation was done using SIFT and Polyphen 2. High-impact effect variants were considered as potentially deleterious or expected to potentially correlate with complete loss of function (LoF) of the affected transcripts [14, 54]. In order to identify rare variants influencing stallion reproduction we filtered high-impact variants within candidate genes for stallion fertility with missing homozygous mutant genotypes in fertile stallions. Under this scenario, we expected that homozygosity for those variants is less likley in fertile stallions. Analyses were performed using SAS/Genetics, version 9.4 (Statistical Analysis System, Cary, NC, 2015).

### Validation population

In total, we analysed 337 fertile stallions of 19 breeds (Additional file 8). The largest proportion of the fertile horses were Hanoverian stallions (*n* = 226), used for artificial insemination at the National State Stud Celle of Lower Saxony in Celle. For 216 Hanoverian stallions breeding values of the paternal component of the pregnancy rate per estrus cycle (EBV-PAT) and de-regressed EBV-PAT were available. The mean reliabilities of the EBVs were at 0.7. For fertile stallions other than Hanoverian, at least 10 viable offspring were reported, targeting those stallions as fertile.

### Validation of high-impact variants

We assumed that the mutant allele with high impact on protein structure should be completely absent or in a very low frequency in fertile stallions. We genotyped high-impact SNPs in fertile stallions using polymerase chain reaction-restriction fragment length polymorphism (PCR-RFLP) and competitive allele specific PCR (KASP) genotyping technique [55] (LGC Genomics GmbH, Berlin, Germany) (Additional file 5). PCR-RFLP primers were designed using Primer3 [56, 57] (http://bioinfo.ut.ee/primer3/). DNA was amplified according to standard protocols on PTC 100 thermal cyclers, MJ Research, Watertown, MA, US. For enzymatic digestion, we used 15 μl reaction volume containing 1.5 μl buffer, 1.5 U endonucleases and 5 μl PCR amplicons. The KASP genotyping reaction was run in 5 μl KASP Master mix 2x (FAM and HEX dye-labeled FRET cassette, *Taq* polymerase, buffer), 0.14 μl KASP Assay mix (KASP-by-design: two allele-specific primers, one common primer) and 5 μl template DNA. Allelic discrimination was carried out using the Applied Biosystems 7300 Real-Time PCR System (Life Technologies).

### Statistical analysis

The ALLELE procedure of SAS/Genetics was used to calculate allele and genotype frequencies, minor allele frequencies (MAF), observed heterozygosity (HET) and χ^2^-tests for Hardy-Weinberg equilibrium for the SNPs genotyped.

Calculation of EBV-PAT in Hanoverians and the model used for estimation are described elsewhere [58]. In brief, data and model employed are given in the following. Fertility data included the breeding seasons 1997 to 2005 of the National State stud Celle of Lower Saxony with a total of 19,897 broadmares, 246 stallions, 199,000 artificial insemination records and 96,114 estrus cycles. Trait analysed was the pregnancy rate per estrus. The trait values were encoded 1 when the artificial insemination (AI) was successful in an estrus cycle otherwise the trait value was 0. The success rate of AIs was verified through the reports of the breeders on the pregnancy of the mare and/or a foaling and/or an abortion. All estrus periods with consecutive AIs in the same breeding season were treated as unsuccessful. In addition, estrus periods reported by the breeder as unsuccessful (missing pregnancy of the mare) were also encoded with a trait value 0. We predicted breeding values (EBVs) for the paternal and embryonic component for each stallion. The animal threshold model for prediction of EBVs included the fixed environmental effects of insemination centre, age of stallion, breeding season, period within breeding season, the number of coverings within breeding season (covering number), time interval between coverings within a estrus (insemination regime), breeding history of mares (previous breeding achievement of mares), the random permanent environmental effects of the mare and stallion and the random additive genetic effects of the stallion and the embryo. The analyses were performed using MTGSAM and Gibbs sampling [59, 60] for estimation of random effects and variance components. Heritability for the paternal component of the pregnancy rate per estrus was 1.1 %. All EBVs were standardized onto a mean of 100 and a standard deviation of 20. The random additive genetic effect of the stallion was defined as the paternal component of the pregnancy rate per estrus (EBV-PAT). EBVs >100 mean higher conception rates of the stallions than the population average. An association analysis for each SNP genotype was performed using the procedure GLM of SAS, version 9.4 (Statistical Analysis System, SAS Institute, Cary, NC, USA). The proportion of variance explained for (de-regressed) EBV-PAT was estimated using the GLM procedure of SAS. In order to account for the population structure a mixed linear model (MLM) was employed using the respective genotype as fixed effect and a random animal effect through an identity-by-state-kinship (IBS) matrix. The IBS matrix reflects the genomic relationship matrix among all individuals genotyped and captures the relatedness among animals as well as the cryptic family structure. We used genotypings from the Equine SNP50 Beadchip (Illumina, San Diego, CA, USA) including 54,602 SNPs. Quality criteria were minor allele frequency (MAF) >0.05, genotyping rate per SNP and animal >0.90 and HWE (*P* < 0.00001). After filtering for quality criteria, 46,074 SNPs remained for building the IBS-matrix [6]. The analysis was run using TASSEL, version 3.0.146 [61]. The Bonferroni correction was calculated using the MULTIPLE TEST procedure of SAS, version 9.4 to determine the threshold for experiment-wide significance.

### Accession numbers

Newly discovered polymorphisms were submitted to NCBI dbSNP database (ss1457622626 -ss1457622648). The whole genome sequences of four stallions, one Arabian, two Hanoverian and one Sorraia are available at the NCBI Sequence Read Archive (SRA) (http://www.ncbi.nlm.nih.gov/sra), accession number SRP033361. Corresponding VCF files can be downloaded at Intrepid Bioinformatics at http://dx.doi.org/10.13013/J6MW2F2B. The whole genome sequences of three stallions, one Icelandic, Standardbred and Przewalski and the re-sequenced reference genome of the Thoroughbred mare ‘Twilight’ can be found at SRA under accession number SRA082086. Corresponding BAM and VCF files can be downloaded at http://geogenetics.ku.dk/publications/middle-pleistocene-omics.

## Additional files

Additional file 1: Gene list of human (gene ID human) and corresponding equine gene identities (mapped gene ID horse) involved in male reproduction processes. Start and end of equine genes in base pairs (bp), chromosomal position of the equine gene (ECA), gene symbol, gene name, gene ontology (GO) term, GO molecular function, GO biological process, GO cellular component and GO pathway are given. (XLSX 5632 kb) Additional file 2: Whole genome sequences of horses filtered for high-impact variants in 11 horses. Type of mutation, single nucleotide polymorphism (SNPs) and Insertions/Deletions (Indels) and type of high-impact effect are given. (XLSX 20 kb) Additional file 3: Equine gene models for 14 genes related with stallion fertility and high-impact variants. Gene models were built based on the Ensembl annotation. Translated exons are shown as solid black boxes, untranslated exons are shown as open boxes. Numbers above the boxes indicate the exon number. Continuous lines represent introns, numbers below the boxes and lines indicate the respective sizes of exons and introns in base pairs. The predicted translation start and stop codon is indicated. The variant with high effect on protein structure, identified by screening whole genome sequences of horses, is given above the exon. (DOCX 734 kb) Additional file 4: Network diagram showing protein and genetic interactions, pathways, co-expression, co-localization and protein domain similarity of 15 genes screened for fertility-related high-impact variants. The genes related with stallion fertility are given in black. (DOCX 503 kb) Additional file 5: Techniques and primer sequences used for genotyping high-impact variants. Competitive allele specific PCR (KASP) genotyping technique was used for genotyping 16/17 variants. Specific FAM and HEX dye labeled primer (Primer_Allele FAM, Primer_Allele HEX) with common primers and annealing temperature (AT) are given. Polymerase chain reaction restriction fragment length polymorphism (PCR-RFLP) was used for 1/17 variants. Primer sequences and position, product size, annealing temperature (AT) and the restriction enzyme used are shown. (DOCX 16 kb) Additional file 6: Distribution of high-impact variants contributing to stallion fertility in discovery breeds compared to validation breeds. The discovery breeds include horses of the breeds Arabian (AR), Hanoverian (HA), Standardbred (SB), Icelandic (IS), Norwegian fjord (NO), and the non-breed horses Dülmen Horse (DU) and Sorraia (SO), and the wild horse Przewalski (PR). The validation breeds include stallions of the pure breeds Arabian (AR), and Thoroughbred (TH), German sport horses including Hanoverian (HA), Holstein (HO), Oldenburg (OL), Westphalian (WE) Rhinelander (RH) and German Riding Pony (GE). Next, cold blood breeds, represented by Black Forest Horses (BF), Mecklenburger Cold Blood (MC), Rhenish German Cold Blood (RG), Saxon Thuringia Cold Blood (ST), Schleswig Cold Blood (SC) and Southern German Cold Blood (SG), further Dülmen Horse (DU) and Sorraia (SO). Further, robust horses and non-breed horses were represented by Norwegian fjord (NO), Konik (KO) and Tarpan (TA). (DOCX 30 kb) Additional file 7: Number of stallions genotyped (n) and genotypic means with their standard errors (SE) and *P*-values (P) of the de-regressed estimated relative breeding values of the paternal component for the pregnancy rate per estrus (EBV-PAT) in 216 Hanoverian stallions for high-impact variants, their nominal (P) and for multiple tests Bonferroni-corrected *P*-values (P_multiple_) for differences among the genotypes. The de-regressed EBV-PAT values were standardized onto a scale of 100 ± 20. (DOCX 20 kb) Additional file 8: Distribution of fertile stallions genotyped for high-impact variants. Fertile stallions have a minimum number of 10 progeny. (DOCX 14 kb)

## Abbreviations

AR: Arabian
BF: Black Forest Horses
CFTR: cystic fibrosis transmembrane conductance regulator
DU: Dülmen Horse
EBV-PAT: estimated breeding values for the paternal component of the pregnancy rate per estrus
FBXO43: F-box only protein 43
FOXP1: forkhead box protein P
GE: German Riding Pony
GHRL: appetite-regulating hormone
GO: gene ontology
HA: Hanoverian
HO: Holstein
HWE: Hardy Weinberg equilibrium
IS: Icelandic
KASP: competitive allele specific PCR
KO: Konik
MAF: minor allele frequency
MC: Mecklenburg Cold Blood
NEURL1: neuralized E3 ubiquitin protein ligase 1
NGS: next-generation sequences
NO: Norwegian fjord
NOTCH1: neurogenic locus notch homolog protein 1
OL: Oldenburg
OVGP1: oviduct-specific glycoprotein
PCR: polymerase chain reaction
PKD1: Polycystin-1
PR: Przewalski
RFLP: restriction fragment length polymorphism
RG: Rhenish Cold Blood
RH: Rhinelander
SB: Standardbred
SC: Schleswig Cold Blood
SG: Southern German Cold Blood
SNP: single nucleotide polymorphism
SO: Sorraia
SPATA31E1: spermatogenesis-associated protein 31E1
ST: Saxon Thuringia Cold Blood
TA: Tarpan
TCP11: t-complex protein 11 homolog
TH: Thoroughbred
TSSK6: testis-specific serine/threonine-protein kinase 6
WE: Westphalian
