# Supplementary material for: Screening of whole genome sequences identified high-impact variants for stallion fertility
Source: BMC Genomics. 2016 Apr 14;17:288. doi: 10.1186/s12864-016-2608-3 (PMC4832559; doi:10.1186/s12864-016-2608-3)

**Additional file 3. Equine gene models for 14 genes related with stallion fertility and high-impact variants.** Gene models were built based on the Ensembl annotation. Translated exons are shown as solid black boxes, untranslated exons are shown as open boxes. Numbers above the boxes indicate the exon number. Continuous lines represent introns, numbers below the boxes and lines indicate the respective sizes of exons and introns in base pairs. The predicted translation start and stop codon is indicated. The variant with high effect on protein structure identified by screening whole genome sequences of horses is given above the exon.


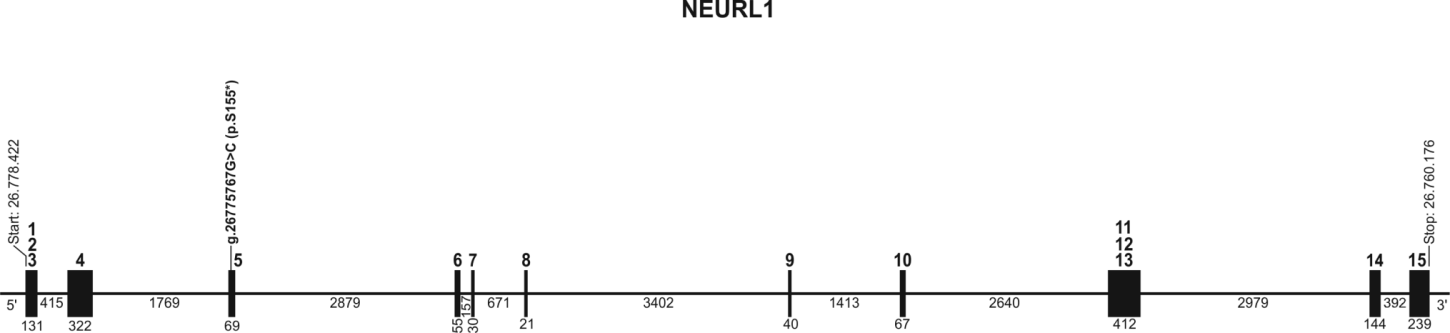


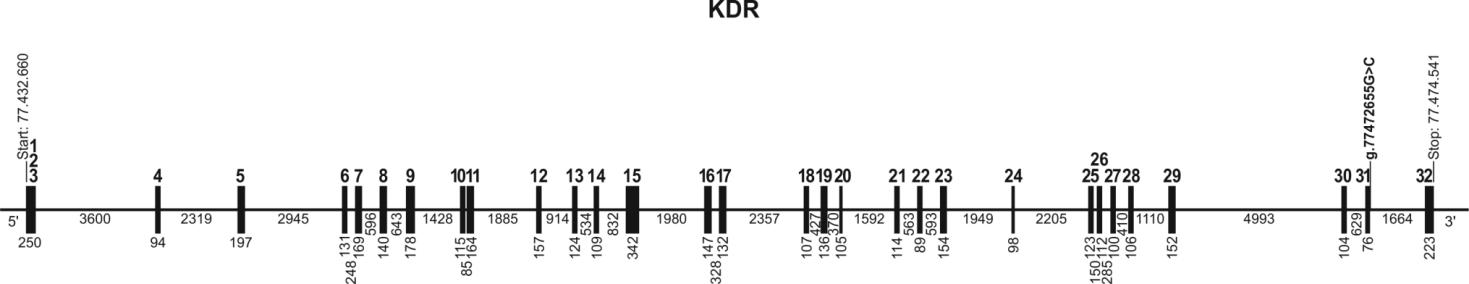


**
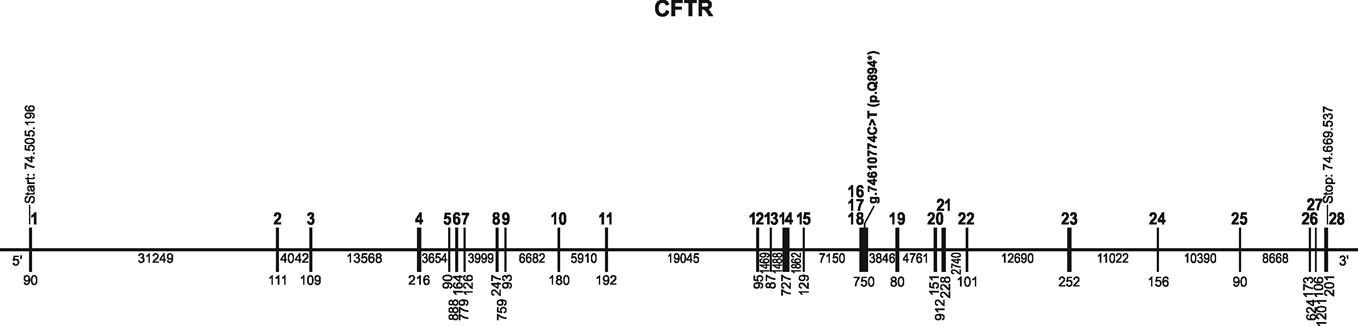
**


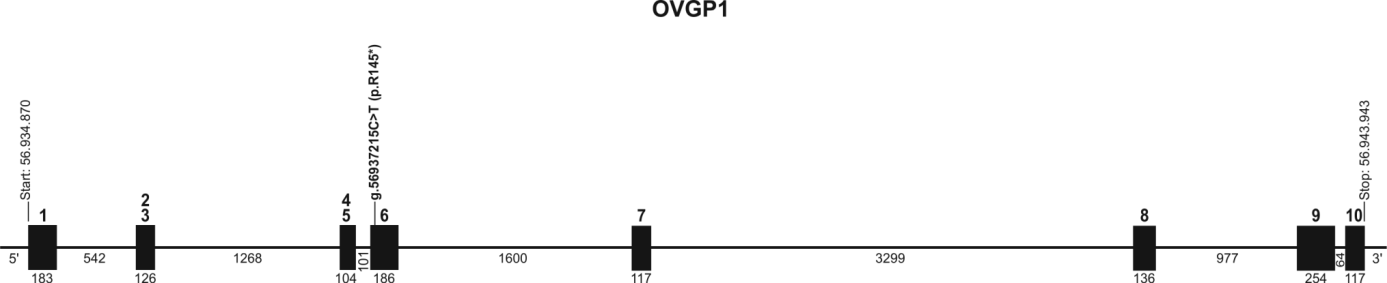


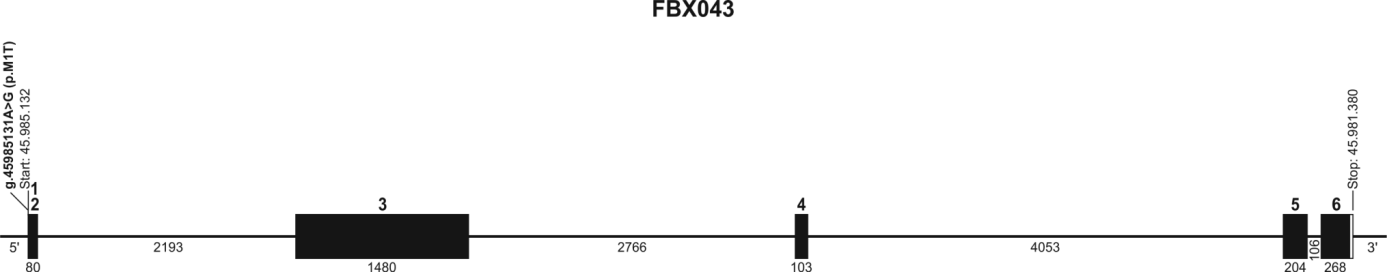


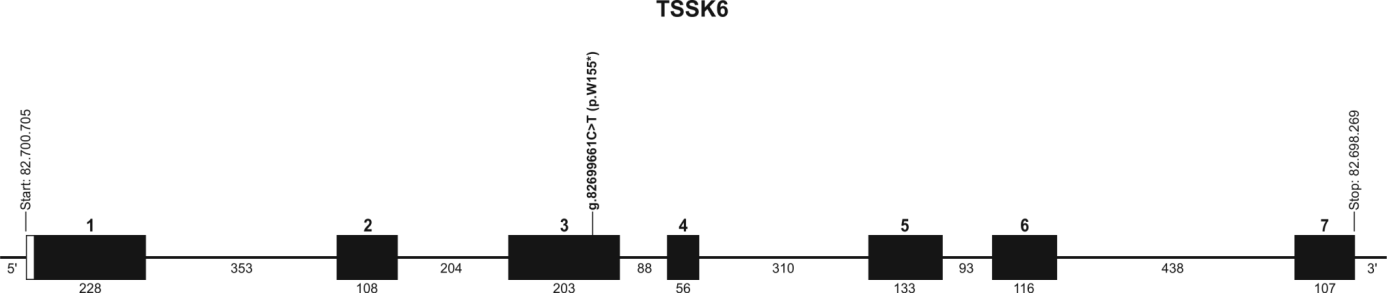


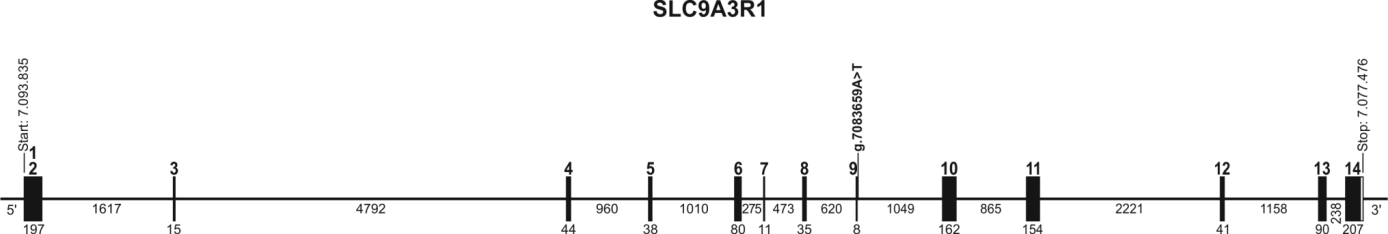


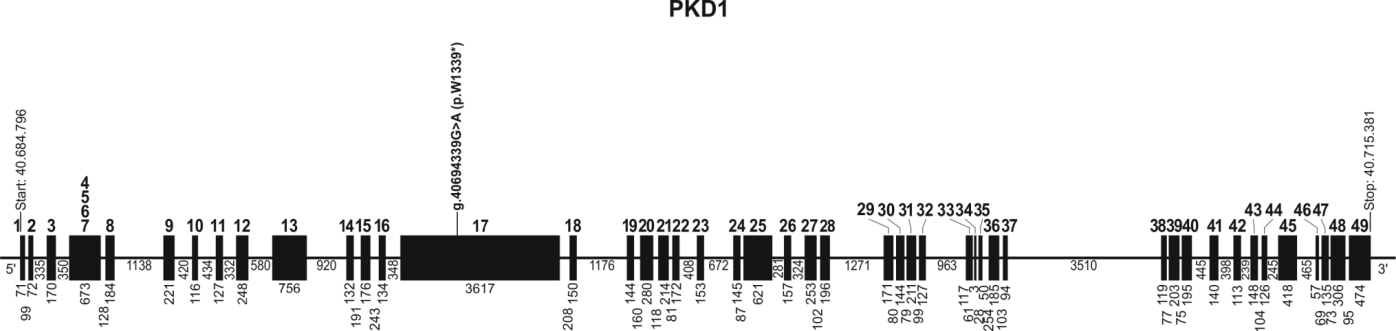


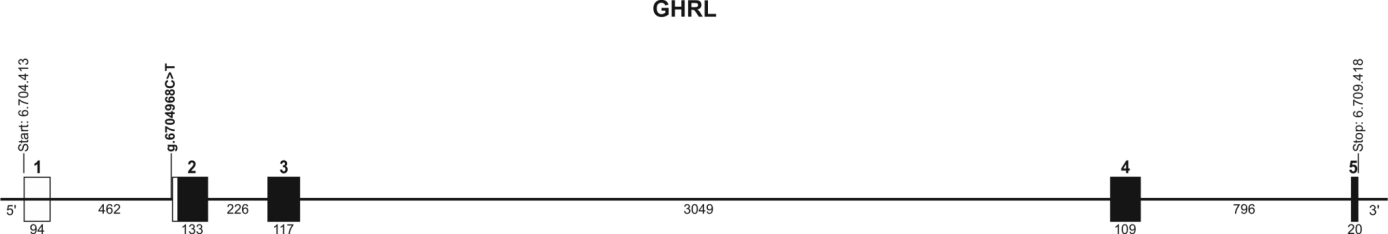


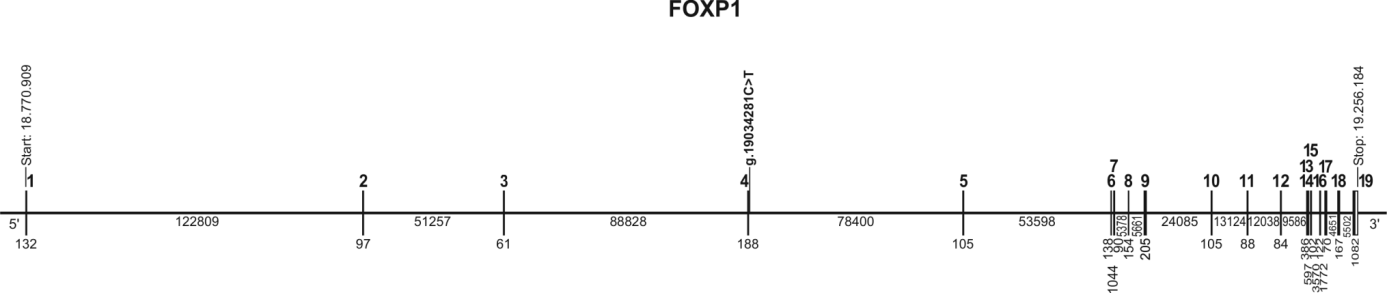


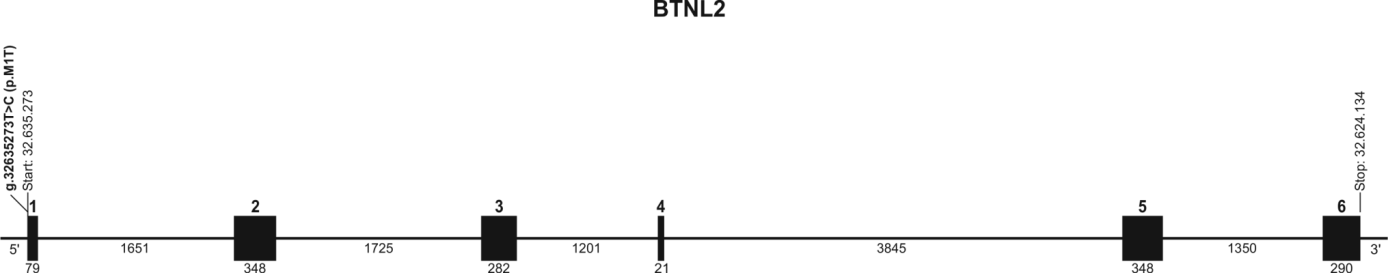


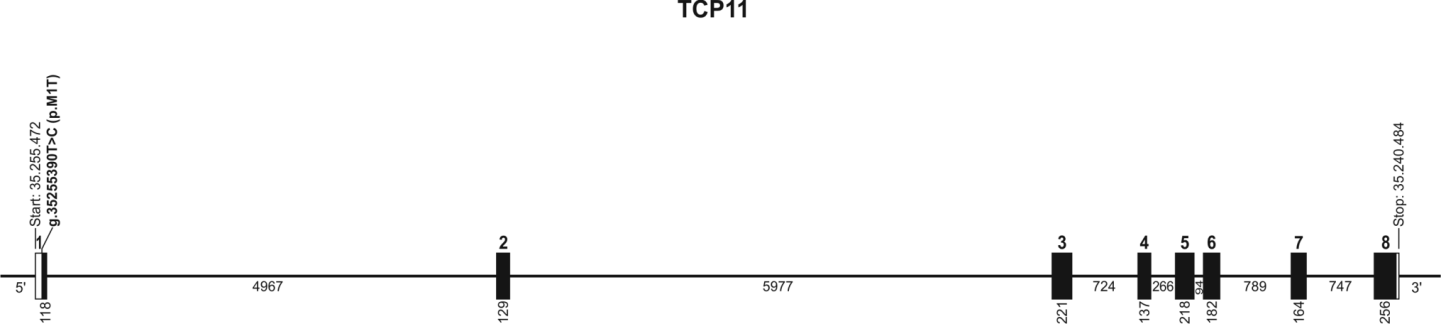


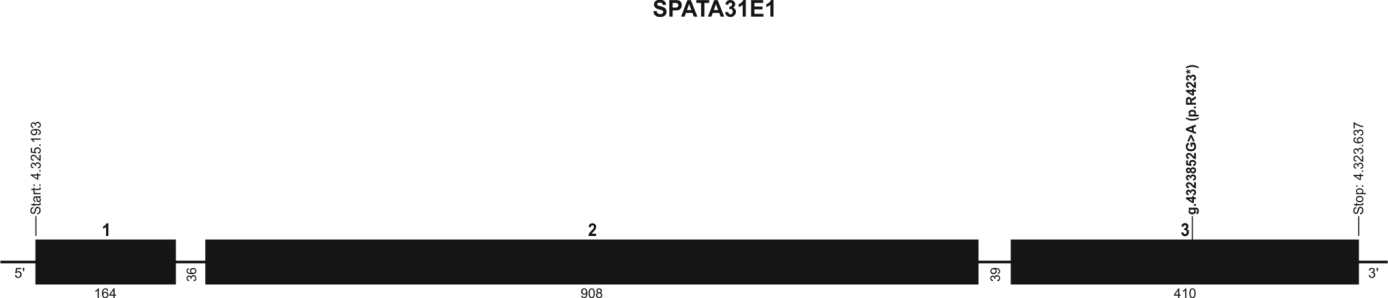


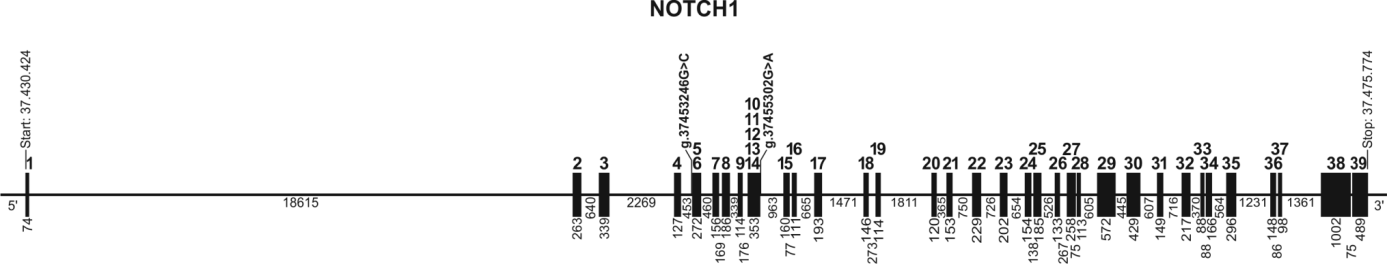

Supplement: Additional file 3: — Equine gene models for 14 genes related with stallion fertility and high-impact variants. Gene models were built based on the Ensembl annotation. Translated exons are shown as solid black boxes, untranslated exons are shown as open boxes. Numbers above the boxes indicate the exon number. Continuous lines represent introns, numbers below the boxes and lines indicate the respective sizes of exons and introns in base pairs. The predicted translation start and stop codon is indicated. The variant with high effect on protein structure, identified by screening whole genome sequences of horses, is given above the exon. (DOCX 734 kb) [file 12864_2016_2608_MOESM3_ESM.docx]
