# Supplementary material for: Screening of whole genome sequences identified high-impact variants for stallion fertility
Source: BMC Genomics. 2016 Apr 14;17:288. doi: 10.1186/s12864-016-2608-3 (PMC4832559; doi:10.1186/s12864-016-2608-3)

**Additional file 4. Network diagram showing protein and genetic interactions, pathways, co-expression, co-localization and protein domain similarity of 15 genes screened for fertility-related high-impact variants.** The genes related with stallion fertility are given in black.


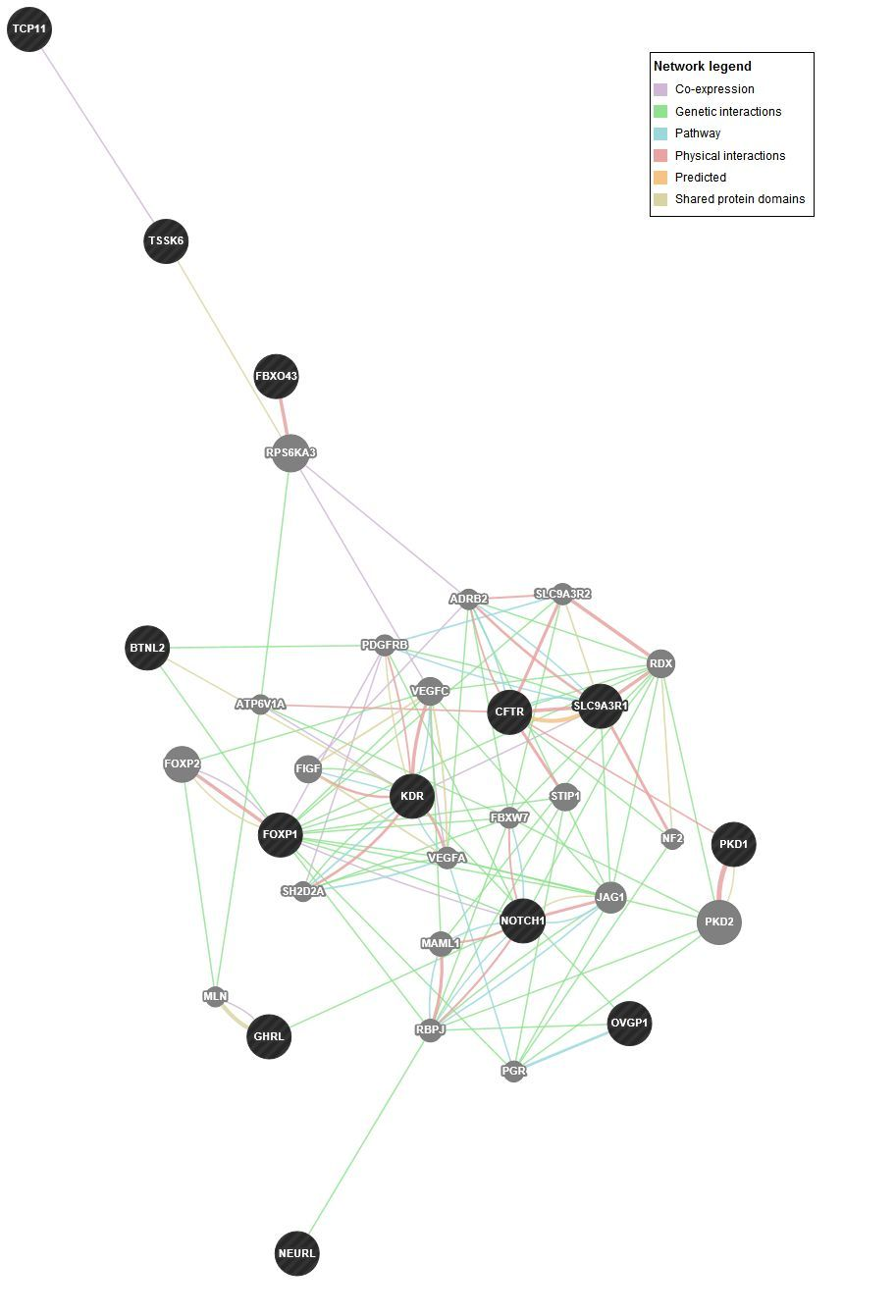

Supplement: Additional file 4: — Network diagram showing protein and genetic interactions, pathways, co-expression, co-localization and protein domain similarity of 15 genes screened for fertility-related high-impact variants. The genes related with stallion fertility are given in black. (DOCX 503 kb) [file 12864_2016_2608_MOESM4_ESM.docx]
