# Supplementary material for: Screening of whole genome sequences identified high-impact variants for stallion fertility
Source: BMC Genomics. 2016 Apr 14;17:288. doi: 10.1186/s12864-016-2608-3 (PMC4832559; doi:10.1186/s12864-016-2608-3)
Supplement: Additional file 5: — Techniques and primer sequences used for genotyping high-impact variants. Competitive allele specific PCR (KASP) genotyping technique was used for genotyping 16/17 variants. Specific FAM and HEX dye labeled primer (Primer_Allele FAM, Primer_Allele HEX) with common primers and annealing temperature (AT) are given. Polymerase chain reaction restriction fragment length polymorphism (PCR-RFLP) was used for 1/17 variants. Primer sequences and position, product size, annealing temperature (AT) and the restriction enzyme used are shown. (DOCX 16 kb) [file 12864_2016_2608_MOESM5_ESM.docx]

**Additional file 5:** **Techniques and primer sequences used for genotyping high-impact variants.** Competitive allele specific PCR (KASP) genotyping technique was used for genotyping 16/17 variants. Specific FAM and HEX dye labeled primer (Primer_Allele FAM, Primer_Allele HEX) with common primers and annealing temperature (AT) are given. Polymerase chain reaction restriction fragment length polymorphism (PCR-RFLP) was used for 1/17 variants. Primer sequences and position, product size, annealing temperature (AT) and the restriction enzyme used are shown.

| **Gene** | **SNP** | **Genotyping** | **Primer Forward (5‘-3‘)** | **Primer reverse (5‘-3‘)** | **Product size (bp)** | **AT °C** |
| --- | --- | --- | --- | --- | --- | --- |
| NEURL1 | g.26775767G>C | PCR-RFLP | TGGGCTAAGGCATAGGACTC | GAAAGGCCAGAGTTACGCTAC | 406 | 59 |
|  |  | (BsmAI) |  |  |  |  |
| **Gene** | **SNP** | **Genotyping** | **Primer_AlleleFAM (5‘-3‘)** | **Primer_AlleleHEX (5‘-3‘)** | **Primer_Common (5‘-3‘)** | **AT °C** |
| KDR | g.77472655G>C | KASP | GCAAATTTGCTCAACCAACAGAGAC | GCAAATTTGCTCAACCAACAGAGAG | TTCAATGTAAGTGCAGAGCTTTCCAGTTT | 64.7 |
| CFTR | g.74610774C>T | KASP | AGTAAATCCTTGAGACTAACGGCAC | ATAGTAAATCCTTGAGACTAACGGCAT | AAGCACAACACATTTCACTAGCAGAGTTT | 64.7 |
| OVGP1 | g.56937215C>T | KASP | GGCATATGCCATGAATTCCTGGC | AGGCATATGCCATGAATTCCTGGT | TCTGGGGGTGCCCCAAGCTTT | 64.6 |
| FBXO43 | g.45985131A>G | KASP | TTTCTTTTTAAGTGCTGTTACTTTGGCAT | CTTTTTAAGTGCTGTTACTTTGGCAC | CCAAACAAGAAAATCTCTGACTGCCTTTA | 6.,9 |
| TSSK6 | g.82699661C>T | KASP | ACAGCGCTGACCAGCTGCC | CACAGCGCTGACCAGCTGCT | GGAGGAGGACGAGGCCCGTA | 63.8 |
| SLC9A3R1 | g.7083659A>T | KASP | GAGGCAGGCAGCGCTAAGAGT | GAGGCAGGCAGCGCTAAGAGA | GAGAGCCCTGGGTTCCTCATTTATA | 65.2 |
| PKD1 | g.40694339G>A | KASP | GTTCTCCTACCGCTACACCTG | CTGTTCTCCTACCGCTACACCTA | CGGGCAACGTCCTTAGTGCCAA | 66.4 |
| GHRL | g.6704968C>T | KASP | ACCCTCTCTCCTCCAGGCC | GACCCTCTCTCCTCCAGGCT | CTTAGCCGGCTGGGGACAGAT | 63.8 |
| FOXP1 | g.19034281C>T | KASP | CCGACGCCGGGGCACAG | CCCGACGCCGGGGCACAA | CACGTGCAGCAGCAGCAGGTA | 64.4 |
| BTNL2 | g.32635273T>C | KASP | CGGTATCCTGGAAGATCCACCAT | GGTATCCTGGAAGATCCACCAC | GTTTCTCTTGTGATTGCTCCAGGGAA | 64.6 |
| TCP11 | g.35255390T>C | KASP | TTCACATGGAAATCGTGACCCAT | TCACATGGAAATCGTGACCCAC | CTGACAGATACAGTTATCGATGTTTCCAA | 64.1 |
| SPATA31E1 | g.4323852G>A | KASP | TGATCCCTGTGAGTGTGCGTC | TGATCCCTGTGAGTGTGCGTT | GCATAGTTGGCAGCAAGCCAGGAT | 64 |
| NOTCH1 | g.37453246G>C | KASP | CCACGTCCTCGGTGCAGTAC | CCACGTCCTCGGTGCAGTAG | TCCCGGCCCCCACAGGTCA | 65.2 |
| NOTCH1 | g.37455302G>A | KASP | CCCTGTCCACCTGGCCTG | GCCCTGTCCACCTGGCCTA | AAGCCCTCTCAGCCATTGTGTCAAT | 63 |
| ENSECAG00000020135 | g.79813487A>T | KASP | GCATCACAATCCCTGACTTCAAACA | GCATCACAATCCCTGACTTCAAACT | GTACCATGCTGTTTTGATTACTGTAGCTT | 64.1 |
| ENSECAG00000021286 | g.25184403G>C | KASP | TTATCCAATCATCTGTTTATGGGCAC | TATCCAATCATCTGTTTATGGGCAG | CAATAGCCAAGACATGGAAGCAACCTA | 65.7 |
