# Supplementary material for: Screening of whole genome sequences identified high-impact variants for stallion fertility
Source: BMC Genomics. 2016 Apr 14;17:288. doi: 10.1186/s12864-016-2608-3 (PMC4832559; doi:10.1186/s12864-016-2608-3)
Supplement: Additional file 6: — Distribution of high-impact variants contributing to stallion fertility in discovery breeds compared to validation breeds. The discovery breeds include horses of the breeds Arabian (AR), Hanoverian (HA), Standardbred (SB), Icelandic (IS), Norwegian fjord (NO), and the non-breed horses Dülmen Horse (DU) and Sorraia (SO), and the wild horse Przewalski (PR). The validation breeds include stallions of the pure breeds Arabian (AR), and Thoroughbred (TH), German sport horses including Hanoverian (HA), Holstein (HO), Oldenburg (OL), Westphalian (WE) Rhinelander (RH) and German Riding Pony (GE). Next, cold blood breeds, represented by Black Forest Horses (BF), Mecklenburger Cold Blood (MC), Rhenish German Cold Blood (RG), Saxon Thuringia Cold Blood (ST), Schleswig Cold Blood (SC) and Southern German Cold Blood (SG), further Dülmen Horse (DU) and Sorraia (SO). Further, robust horses and non-breed horses were represented by Norwegian fjord (NO), Konik (KO) and Tarpan (TA). (DOCX 30 kb) [file 12864_2016_2608_MOESM6_ESM.docx]

**Additional file 6.** Distribution of high-impact variants contributing to stallion fertility in discovery breeds compared to validation breeds. The discovery breeds include horses of the breeds Arabian (AR), Hanoverian (HA), Standardbred (SB), Icelandic (IS), Norwegian fjord (NO), and the non-breed horses Dülmen Horse (DU) and Sorraia (SO), and the wild horse Przewalski (PR). The validation breeds include stallions of the pure breeds Arabian (AR), and Thoroughbred (TH), German sport horses including Hanoverian (HA), Holstein (HO), Oldenburg (OL), Westphalian (WE) Rhinelander (RH) and German Riding Pony (GE). Next, cold blood breeds, represented by Black Forest Horses (BF), Mecklenburger Cold Blood (MC), Rhenish German Cold Blood (RG), Saxon Thuringia Cold Blood (ST), Schleswig Cold Blood (SC) and Southern German Cold Blood (SG), further Dülmen Horse (DU) and Sorraia (SO). Further, robust horses and non-breed horses were represented by Norwegian fjord (NO), Konik (KO) and Tarpan (TA).

| SNP IDs | Discovery breeds | | | | | | | | Validation breeds | | | | | | | | | | | | | | | | | | |
| --- | --- | --- | --- | --- | --- | --- | --- | --- | --- | --- | --- | --- | --- | --- | --- | --- | --- | --- | --- | --- | --- | --- | --- | --- | --- | --- | --- |
|  |  |  |  |  |  |  |  |  | Pure breed | | German sport horse | | | | |  | Cold blood | | | | | | Robust/Non-breed | | | | |
|  | AR | HA | SB | IS | NO | DU | SO | PR | AR | TH | HA | HO | OL | WE | RH | GE | BF | MC | RG | ST | SC | SG | NO | DU | SO | KO | TA |
| g.26775767G>C |  | **+** |  |  |  | **+** |  |  |  | | **+** |  |  | + |  |  |  |  | + |  | + | + |  | **+** |  |  | + |
| NEURL1 |  |  |  |  |  |  |  |  |  | |  |  |  |  |  |  |  |  |  |  |  |  |  |  |  |  |  |
| g.77472655G>C |  | **+** |  |  |  |  | **+** |  | + | + | **+** | + |  | + | + |  | + | + |  |  |  |  |  | + | **+** | + |  |
| KDR |  |  |  |  |  |  |  |  |  |  |  |  |  |  |  |  |  |  |  |  |  |  |  |  |  |  |  |
| g.74610774C>T |  |  |  |  |  |  |  | + |  |  | + |  |  |  |  |  |  |  |  |  |  |  |  |  |  |  |  |
| CFTR |  |  |  |  |  |  |  |  |  |  |  |  |  |  |  |  |  |  |  |  |  |  |  |  |  |  |  |
| g.56937215C>T |  | **+** |  |  |  |  |  |  |  | + | **+** |  |  | + |  |  |  |  |  |  |  |  |  |  |  |  |  |
| OVGP1 |  |  |  |  |  |  |  |  |  | |  |  |  |  |  |  |  |  |  |  |  |  |  |  |  |  |  |
| g.45985131A>G |  |  |  |  |  | + |  |  | + |  | + |  |  | + |  |  |  |  |  |  |  |  |  |  |  |  |  |
| FBXO43 |  |  |  |  |  |  |  |  |  |  |  |  |  |  |  |  |  |  |  |  |  |  |  |  |  |  |  |
| g.82699661C>T |  |  |  |  |  | **+** |  |  | + |  | + | + |  |  |  | + | + | + |  |  |  |  |  | **+** | + |  |  |
| TSSK6 |  |  |  |  |  |  |  |  |  |  |  |  |  |  |  |  |  |  |  |  |  |  |  |  |  |  |  |
| g.7083659A>T |  | **+** | + |  |  |  |  |  | + | + | **+** | + | + | + | + |  | + |  | + |  |  |  | + |  |  | + |  |
| SLC9A3R1 |  |  |  |  |  |  |  |  |  |  |  |  |  |  |  |  |  |  |  |  |  |  |  |  |  |  |  |
| g.40694339G>A |  |  |  | + |  |  |  |  |  |  | + |  |  |  |  |  |  |  |  |  |  |  |  |  |  |  |  |
| PKD1 |  |  |  |  |  |  |  |  |  |  |  |  |  |  |  |  |  |  |  |  |  |  |  |  |  |  |  |
| g.6704968C>T |  |  |  |  | **+** |  |  |  |  |  | + |  |  |  |  |  | + |  |  | + |  |  | **+** |  |  |  | + |
| GHRL |  |  |  |  |  |  |  |  |  |  |  |  |  |  |  |  |  |  |  |  |  |  |  |  |  |  |  |
| g.19034281C>T |  |  |  |  |  | **+** |  |  | + |  | + | + |  | + | + |  | + | + | + | + |  |  |  | **+** |  |  | + |
| FOXP1 |  |  |  |  |  |  |  |  |  |  |  |  |  |  |  |  |  |  |  |  |  |  |  |  |  |  |  |
| g.32635273T>C |  | **+** |  |  | + |  |  |  |  | + | **+** | + |  | + |  |  | + |  | + |  |  |  |  |  |  |  |  |
| BTNL2 |  |  |  |  |  |  |  |  |  |  |  |  |  |  |  |  |  |  |  |  |  |  |  |  |  |  |  |
| g.35255390T>C |  |  |  | + |  |  |  |  |  |  | + |  |  |  |  |  |  |  |  |  |  |  |  |  |  |  |  |
| TCP11 |  |  |  |  |  |  |  |  |  |  |  |  |  |  |  |  |  |  |  |  |  |  |  |  |  |  |  |

| SNP IDs | Discovery breeds | | | | | | | | Validation breeds | | | | | | | | | | | | | | | | | | |
| --- | --- | --- | --- | --- | --- | --- | --- | --- | --- | --- | --- | --- | --- | --- | --- | --- | --- | --- | --- | --- | --- | --- | --- | --- | --- | --- | --- |
|  |  |  |  |  |  |  |  |  | Pure breed | | German sport horse | | | | |  | Cold blood | | | | | | Robust/Non-breed | | | | |
|  | AR | HA | SB | IS | NO | DU | SO | PR | AR | TH | HA | HO | OL | WE | RH | GE | BF | MC | RG | ST | SH | SG | NO | DU | SO | KO | TA |
| g.4323852G>A |  |  |  |  | + |  |  |  |  | |  | + |  |  |  |  |  |  |  |  |  | + |  |  |  |  |  |
| SPATA31E1 |  |  |  |  |  |  |  |  |  | |  |  |  |  |  |  |  |  |  |  |  |  |  |  |  |  |  |
| g.37453246G>C | **+** |  |  |  |  |  |  |  | **+** |  | + |  |  |  | + |  |  |  |  | + | + |  |  |  |  |  |  |
| NOTCH1 |  |  |  |  |  |  |  |  |  |  |  |  |  |  |  |  |  |  |  |  |  |  |  |  |  |  |  |
| g.37455302G>A |  | **+** |  |  |  |  | + |  | + | + | **+** | + |  |  | + |  | + | + |  | + | + |  |  |  |  |  |  |
| NOTCH1 |  |  |  |  |  |  |  |  |  |  |  |  |  |  |  |  |  |  |  |  |  |  |  |  |  |  |  |
| g.79813487A>T |  |  |  | + |  |  |  |  |  |  |  |  |  |  |  |  |  |  |  |  |  |  |  |  |  |  |  |
| ENSECAG00000020135 |  |  |  |  |  |  |  |  |  | |  |  |  |  |  |  |  |  |  |  |  |  |  |  |  |  |  |
| g.25184403G>C |  |  |  | + |  |  |  |  |  |  |  |  |  |  |  |  |  |  |  |  |  |  |  |  |  |  |  |
| ENSECAG00000021286 |  |  |  |  |  |  |  |  |  |  |  |  |  |  |  |  |  |  |  |  |  |  |  |  |  |  |  |
