# Supplementary material for: Screening of whole genome sequences identified high-impact variants for stallion fertility
Source: BMC Genomics. 2016 Apr 14;17:288. doi: 10.1186/s12864-016-2608-3 (PMC4832559; doi:10.1186/s12864-016-2608-3)
Supplement: Additional file 7: — Number of stallions genotyped (n) and genotypic means with their standard errors (SE) and P-values (P) of the de-regressed estimated relative breeding values of the paternal component for the pregnancy rate per estrus (EBV-PAT) in 216 Hanoverian stallions for high-impact variants, their nominal (P) and for multiple tests Bonferroni-corrected P-values (Pmultiple) for differences among the genotypes. The de-regressed EBV-PAT values were standardized onto a scale of 100 ± 20. (DOCX 20 kb) [file 12864_2016_2608_MOESM7_ESM.docx]

**Additional file 7. Number of stallions genotyped (n) and genotypic means with their standard errors (SE) and P-values (P) of the de-regressed estimated relative breeding values of the paternal component for the pregnancy rate per estrus (EBV-PAT) in 216 Hanoverian stallions for high-impact variants, their nominal (P) and for multiple tests Bonferroni-corrected P-values (P_multiple_) for differences among the genotypes.** The de-regressed EBV-PAT values were standardized onto a scale of 100±20.

| SNP ID | GT | EBV- PAT | SE | P | P_multiple_ |
| --- | --- | --- | --- | --- | --- |
| g.26775767G>C | G/G | 101 | 1.11 | 0.295 | 1.0 |
| NEURL1 | G/C | 97 | 2.85 |  |  |
| g.77472655G>C  KDR | G/G | 100 | 1.18 | 0.164 | 1.0 |
|  | G/C | 101 | 2.16 |  |  |
|  | C/C | 114 | 7.34 |  |  |
| g.74610774C>T | C/C | 101 | 1.07 | 0.994 | 1.0 |
| CFTR | C/T | 102 | 15.03 |  |  |
| g.56937215C>T | C/C | 101 | 0.99 | 0.161 | 1.0 |
| OVGP1 | C/T | 97 | 2.71 |  |  |
| g.45985131A>G | A/A | 101 | 1.04 | 0.275 | 1.0 |
| FBXO43 | A/G | 95 | 5.57 |  |  |
| g.82699661C>T | C/C | 101 | 1.03 | 0.770 | 1.0 |
| TSSK6 | C/A | 103 | 7.35 |  |  |
| g.7083659A>T  SLC9A3R1 | A/A | 99 | 1.50 | 0.474 | 1.0 |
|  | A/T | 101 | 1.59 |  |  |
|  | T/T | 103 | 2.95 |  |  |
| g.40694339G>A | G/G | 100 | 1.04 | 0.950 | 1.0 |
| PKD1 | G/A | 101 | 8.55 |  |  |

| SNP ID | GT | EBV- PAT | SE | P |  |
| --- | --- | --- | --- | --- | --- |
| g.6704968C>T | C/C | 101 | 1.03 | 0.992 | 1.0 |
| GHRL | C/T | 100 | 10.45 |  |  |
| g.19034281C>T | C/C | 101 | 1.06 | 0.07 | 0.98 |
| FOXP1 | C/T | 93 | 4.08 |  |  |
| g.32635273T>C  BTNL2 | C/C | 101 | 1.97 | 0.607 | 1.0 |
|  | C/T | 102 | 2.11 |  |  |
|  | T/T | 100 | 1.39 |  |  |
| g.35255390T>C | T/T | 102 | 1.06 | 0.921 | 1.0 |
| TCP11 | T/C | 100 | 5.60 |  |  |
| g.4323852G>A | G/G | 101 | 1.03 | 0.667 | 1.0 |
| SPATA31E1 | G/A | 96 | 10.45 |  |  |
| g.37453246G>C | G/G | 101 | 1.06 | 0.642 | 1.0 |
| NOTCH1 | G/C | 105 | 10.56 |  |  |
| g.37455302G>A  NOTCH1 | G/G | 101 | 1.10 | 0.00003 | 0.00045 |
|  | G/A | 100 | 2.03 |  |  |
|  | A/A | 60 | 8.04 |  |  |
| g.79813487A>T |  |  |  | na | na |
| ENSECAG00000020135 | A/A | 100 | 2.88 |  |  |
| g.25184403G>C |  |  |  | na | na |
| ENSECAG00000021286 | G/G | 100 | 2.88 |  |  |
