# Supplementary material for: Screening of whole genome sequences identified high-impact variants for stallion fertility
Source: BMC Genomics. 2016 Apr 14;17:288. doi: 10.1186/s12864-016-2608-3 (PMC4832559; doi:10.1186/s12864-016-2608-3)
Supplement: Additional file 8: — Distribution of fertile stallions genotyped for high-impact variants. Fertile stallions have a minimum number of 10 progeny. (DOCX 14 kb) [file 12864_2016_2608_MOESM8_ESM.docx]

**Additional file 8. Distribution of fertile stallions genotyped for high-impact variants.** Fertile stallions have a minimum number of 10 progeny.

| Breed | Number of stallions |
| --- | --- |
| Arabian | 17 |
| Black Forest Horse | 20 |
| Dülmen Horse | 9 |
| Norwegian fjord | 1 |
| German Riding Pony | 1 |
| Hanoverian | 226 |
| Holstein | 6 |
| Konik | 2 |
| Mecklenburg Cold Blood | 7 |
| Oldenburg | 1 |
| Rhenish German Cold Blood | 7 |
| Rhinelander | 1 |
| Saxon Thuringia Cold Blood | 8 |
| Schleswig Cold Blood | 7 |
| Sorraia | 1 |
| South German Cold Blood | 1 |
| Tarpan | 1 |
| Thoroughbred | 11 |
| Westphalian | 10 |
| Total fertile stallions | 337 |
